# Quantifying water stress of safflower (*Carthamus tinctorius* L.) cultivars by crop water stress index under different irrigation regimes

**DOI:** 10.1016/j.heliyon.2022.e09010

**Published:** 2022-02-24

**Authors:** Ehsan Bijanzadeh, Seyed Mojtaba Moosavi, Fatemeh Bahadori

**Affiliations:** a Associate Professor of Agroecology Department, College of Agriculture and Natural Resources of Darab, Shiraz University, Iran; b Former Graduate Student of Agroecology Department, College of Agriculture and Natural Resources of Darab, Shiraz University, Iran

**Keywords:** Vapor pressure deficit, Relative water content, Color quality, Water use efficiency

## Abstract

Infrared thermometry allows evaluating water status of the crop by measuring crop water stress index (CWSI), without the need of physical contact to leaves. In order to quantify water stress by CWSI and finding the best irrigation regime a two-year field experiment was conducted in safflower during 2017 and 2018 growing seasons at Darab, Fars province, Iran. Two safflower cultivars (Goldasht and Local Isfahan) and four irrigation regimes consisted of well-watered [Irrigation based on 100% field capacity (FC)], mild (75% FC), severe (50% FC), and most severe (25% FC) water stress were arranged as split plot according to randomized completely block design with four replicates. The relationship between vapor pressure deficit (VPD) and canopy-air temperature differences (Tc-Ta) was plotted under upper (fully stressed) and lower baselines (non-stressed) equations. In two cultivars, by VPD increment, the distance between upper and lower base lines increased. In Goldasht, the upper baseline (Tc-Ta)ul, was 7.8 °C in 2017 and 8.9 °C in 2018. From April to July when air warmed, Tc-Ta differential was increased up to July and the highest seasonal CWSI (0.72–0.77) were obtained in Local Isfahan under most severe water stress. In 2017, under water stress, the highest relative water content (RWC; 55%), color quality (6–7) and water use efficiency (WUE; 2.69 g m^−2^ mm^−1^) was observed in Goldasht under mild water stress which was more than 2018 and Local Isfahan. It might be attributed the more tolerance of Goldasht to water stress and lower air temperature and evaporation in the first year. CWSI with total water consumed (R^2^ = 0.88∗∗), RWC (R^2^ = 0.87∗∗), color quality (R^2^ = 0.75∗) and seed yield (R^2^ = 0.83∗∗) related, negatively. Overall, a mild water stress (75% FC) with 0.28–0.33 seasonal CWSI had higher RWC, color quality, WUE, with an acceptable yield, which could be the best irrigation regime under water deficit conditions for safflower.

## 1 Introduction

Safflower (*Carthamus tinctorius* L.) is a main crop in arid and semiarid fields of the world because of water stress limitations and cultured in marginal lands which are dry, periodically (McPherson et al., 2004). In recent years, the importance of safflower as oil crop has increased, mainly with the interest in biofuels production (Sajedi et al., 2012; Bonfim-Silva et al., 2015). The high volume of oil imports in Iran due to shortage of oilseeds production and the limitation of water resources, indicated the necessity of identifying species adapted to water stress conditions (Tabib Loghmani et al., 2019). Safflower can develop under high salinity, temperatures, and water stress conditions, and cultured for flowers and used for flavor, medicinal characteristics, coloring, and fodder. The safflower seed has good quality with high oil contents (35%–40%) (Zareie et al., 2013; Torabi et al., 2015).

To food security, water stress is the main critical threat mainly in terrestrial ecosystems of the world. The level of water stress is unpredictable and depended on some factors including distribution and occurrence precipitation, water storing capacity of soils and evaporative demands. Vegetative and reproductive stages of safflower are sensitive to water stress (Koutroubas et al., 2009; Bijanzadeh et al., 2013). These main developmental stages are affected by some physiological reactions, which may decrease crop growth and seed yield. Water shortage during the reproductive stage severely influenced safflower production in comparison to normal irrigation. Likewise, leaf water potential and leaf relative water content (RWC) of safflower were affected by water stress, negatively while antioxidant compound and lipid peroxidation values were increased (Hojati et al., 2011; Pasban Eslam, 2011; Sajedi et al., 2012). The onset of dry and hot conditions during the seed filling phase affected the photosynthesis rate, dry matter remobilization, and the sink size of safflower seeds. The abiotic stresses such as water stress decline photosynthesis and crop nutrient uptake which caused decreasing of safflower seed yield (Koutroubas and Papakosta, 2010). Water stress in the soil during the flowering to seed filling of safflower, declined yield components and yield, negatively (Movahhedy Dehnavy et al., 2009; Koutroubas et al., 2009; Tabib Loghmani et al., 2019).

Nowadays, the demands for freshwater are increasing due to increment the human activity and population (Lei et al., 2016). The suitable knowledge of crop reaction to abiotic stresses such as water stress is necessary to demand the crop production (Behmann et al., 2014). Measurement of stomatal conductance and leaf water potential are the main indices to evaluate the crop water status but their measurements are destructive and labor intensive. Therefore, automated methods for tracing crop water status which can prepare fast and non-destructive evaluation, are required (Pasban Eslam, 2011; Parkash and Singh, 2020).

Innovation in recognize of crop water stress throughout growing season to decline damage of biological yield production, seed yield loss and water use optimization is required. Crop water stress index (CWSI) is a remotely sensed crop stress index based on the infrared spectral areas, which have high accuracy, low time and cost (Irandoust and Bijanzadeh, 2018; Parkash and Singh, 2020). CWSI concept is according to the fact that leaf transpiration cools the canopy and as humidity of rhizosphere is diminished, leaf transpiration and stomatal conductance declined and canopy temperature enhanced (Bijanzadeh et al., 2019; Parkash and Singh, 2020). The CWSI application gained popularity when Idso et al. (1981) showed a linear relationship between Tc-Ta (canopy and air temperature differential) with vapor pressure deficit (VPD), and introduced an empirical method to quantify crop water stress. In safflower grown in areas with dry and hot climate in spring and summer, a disadvantage is that regardless of sowing time, reproductive stage falls into end of spring and early of summer when evapotranspiration values increased, sharply and rainfall stopped (Koutroubas et al., 2009). In sought of Iran, water is a rare resource because of deficit rainfall especially in spring and summer. Monitoring the water status based on a suitable method had a main role to conserve the water resources and developing sustainable agriculture (Torabi et al., 2015; Tabib Loghmani et al., 2019). In Iran, safflower is usually grown at the same time as wheat and canola and little information has been published in terms of CWSI assessment of safflower compared to the other crops. Bijanzadeh and Emam (2012) suggested that under excess and well water treatments, mean CWSI in wheat cultivars was in range of 0.31–0.36, and water deficit in the soil resulted in CWSI increment. Argyrokastritis et al. (2015) reported that in cotton with respect to CWSI value, applying a deficit irrigation program by 50% of water available had significant effect for water saving in the field. Also, Ramirez et al. (2016) declared that, reaching the mean CWSI amounts lower than 0.4, is the best target for irrigation scheduling in potato under drought stress conditions.

In the south of Iran farmers irrigate safflower in the spring and summer and apply a few supplemental irrigations due to cut-off rainfall from April to July (Sajedi et al., 2012). The CWSI related to canopy-air temperature difference has been approved largely as an index to describe crop water amount and irrigation scheduling in some plants and a suitable irrigation regime needs to be planned for every area where rainfall is scarce (Ayed et al., 2017; Alghory and Yazar, 2019; Bijanzadeh et al., 2019). We hypothesized that use of CWSI by quantifying the water stress of safflower under different irrigation regimes would benefit for water management, especially in semi-arid areas. The main goal of the current experiment was quantifying water stress by CWSI calculation and finding the best irrigation regime with respect to CWSI amount in two safflower cultivars under late season water stress.

## 2 Materials and methods

### 2.1 Site description

To evaluate the CWSI for tracing water status of safflower a two-year field experiment was conducted during December 2017 to July 2018 and December 2018 to July 2019 at the Research Station of College of Agriculture and Natural Resources of Darab (28° 75′ 11.14ʺ N, 54° 44′ 78.87ʺ E), Darab, Fars province, Iran. The soil type in the experiment site was loam (fine, loamy, carbonatic, hyperthermic, typic Torriorthents) and the other soil characteristics are given in Table 1. The used soil was classified as Calcaric Regosols (IUSS Working Group WRB, 2015). The study area has a semi-arid climate with cool and rainy winters and dry and hot summers. Also, some monthly climatic data for the research field during 2017–18 and 2018-19 growing seasons are given in Table 2.

**Table 1:** Physical and chemical characteristics of the soil in the experimental site.

Soil depth (cm)	Sand (%)	Silt (%)	Clay (%)	Organic content (%)	Electrical conductivity (dS m^−1^)	pH	N (%)	P (mg kg^−1^)	K (mg kg^−1^)	Field capacity (FC) (cm^3^cm^−3^)	Permanent wilting poit (PWP) (cm^3^cm^−3^)
0–30	38.12	44.0	17.88	0.977	1.092	7.42	0.084	54	320	0.21	0.07
30–60	38.63	44.11	17.26	0.970	1.090	7.54	0.084	58	311	0.21	0.07
60–90	39.80	43.67	16.53	0.84	1.088	7.71	0.083	57	301	0.20	0.08

**Table 2:** Minimum and maximum air temperatures, monthly rainfall, and pan evaporation of the experimental siten in 2017–2018 and 2018–2019 growing seasons.

Month	Temperature (ºC)						Raifall (mm)		Pan evaporation (mm)	
	2017–18			2018–19			2017–18	2018–19	2017–18	2018–19
	Min	Max	Mean	Min	Max	Mean				
December	5.1	20.1	12.6	5.7	21.2	13.5	2.2	24.8	77.6	88.4
January	4.4	19.2	11.8	3.8	23.4	13.6	86.4	94.2	66.3	78.5
February	8.6	21.4	15.0	9.2	23.6	17.9	0	57.9	88.1	91.2
March	10.5	25.4	18.0	8.1	28.5	19.3	47.8	103.4	143.1	175.3
April	12.9	29.8	21.4	8.9	33.2	22.6	0	4.4	202.3	218.9
May	17.9	34.4	26.2	15.8	38.1	30.0	0	0.0	276.2	297.4
June	22.4	39.7	31.1	20.3	42.4	32.9	0	0.0	301.7	345.7
July	22.8	42.7	32.7	24.3	44.2	34.2	0	0.0	344.1	376.3

### 2.2 Experimental details and treatments

The plot size was 3 m × 5 m and which was surrounded with a 25 cm high earth berm, by a 1m wide buffer space between the plots. In the both years, two safflower cultivars including Goldasht and Local Isfahan were cultured as sub plot under irrigation regimes as main plot. Four Irrigation regimes included well-watered [Irrigation based on 100% field capacity (FC)], mild water stress (75% FC), severe water stress (50% FC), and most severe water stress (25% FC). Also, there was an unirrigated plot for each cultivar to evaluate the upper baseline (fully stressed) needed to CWSI evaluation. Overall, 10 plots were designed in each replication and the treatments were arranged as split plot according to randomized completely block design with four replicates. Goldasht is a dwarf cultivar and thornless, while the local Isfahan is a tall and thorny cultivar. Seedbed was prepared by mouldboard ploughing and disking. After the seedbed preparation, uniform safflower seeds were sown by hand at a soil depth of 3 cm in rows 50 cm apart, giving 40 plants m ^−2^ planting density. Safflower seeds were sown on December 6^th^ 2017, and December 5^th^ 2018. By cut off effective rainfall in the Apirl (Table 2), irrigation regimes were started which is synchronized with capitulum emergence stage [Stage 59; based on BBCH scale illustrated by Flemmer et al. (2015)]. Finally, plants in the area of 1 m^2^ from central rows were hand-harvested at each treatment, on 19 July 2018 and 17 July 2019 for seed yield assessment. Based on soil test, only 100 kg ha^−1^ nitrogen fertilizer was used as urea (46% nitrogen) source in each plot at three splits; i.e., one third used before sowing, one third at branching stage [stage 20; Flemmer et al. (2015)], and the rest at the stem elongation stage (stage 39) in all irrigation regimes.

**Figure 1:**
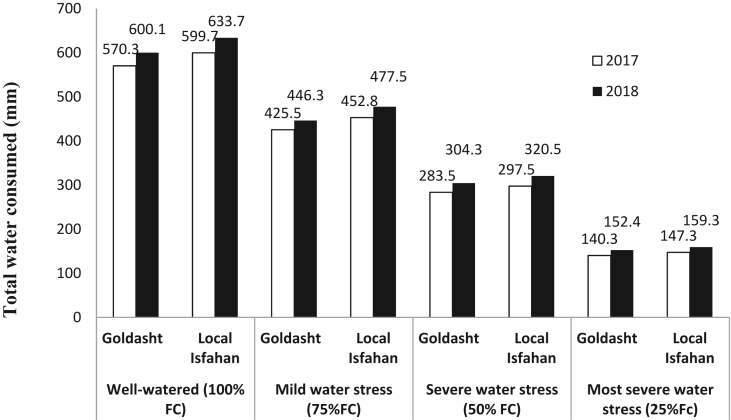
Total water consumed (mm) in each irrigation regime and safflower cultivar during the two growing seasons.

The soil water content was measured in each plot at 30 cm depth down to 90 cm, gravimetrically. Before each irrigation, the soil profile was sampled up to 90-cm by an auger and the water consumed in well-watered irrigation was based on restoring root zone moisture deficit (when 50% of available water was depleted in depth of 90 cm) to near-field capacity. The water amount consumed in each plot was determined by time-volume technique. In this technique water is used by polyethylene pipes placed in each plot and the time of irrigation is calibrated with a timer and a standard container and irrigation water amount of plot (determined by gravimetric technique) was converted to time (min) (Grimes et al., 1987; Barati and Ghadiri, 2017). Total water consumed (mm) (irrigation amount + ranifall) in each irrigation regime and safflower cultivars during the two growing seasons were given in Figure 1.

The irrigation water depth of the soil layers was calculated using Eq. (1).

(1) $$D=\overset{n}{\underset{i=1}{\sum }}\left(\theta \text{fci}-\theta i\right)\Delta Zi$$

where, D is the irrigation water depth (mm), *i* is equal to one layer, *n* is the number of the soil layer, Σ is the summation of the amount of irrigation water depth (mm) in the *n* layer, θfci is the volumetric water content at the field capacity (cm^3^.cm^−3^) in the *i*^th^ soil layer, θi is the volumetric water content (cm^3^ cm^−3^) in the *i*^th^ soil layer and ΔZ is the soil layer thickness (mm) in the *i*^th^ soil layer. A surface drip irrigation system was applied for irrigation. A 20 mm diameter polyethylene pipe with in-line drippers at 40 cm intervals was placed on one side of each safflower row.

### 2.3 Upper and lower baselines and CWSI measurement

Infrared thermometer (LT Lutron, Model TM-958, Taiwan) is a hand-hold and non-contact temperature instrument which measures the temperature in range of -30 to 300 °C (−22 to 572℉) by 0.5 °C or 0.5℉ resolution. It's equipped with spectral band filter of a 9–14 μm and an air temperature sensor. The emissivity value of measurement target set to 0.95. The weight of this model is 140 g, which auto data hold, auto power off and emissivity adjustment. This instrument was applied to determine the canopy temperature after 3 days in each irrigation regime from April to July 2018 and 2019 for the first and second years of the experiment. The irrigation intervals were between 7 to 9 days from April to late season with respect to cultivar type and irrigation regime. To collect the correct data, the infrared thermometer was kept with an angle of 45°, at a height of 1.5 m above the ground during the evaluations Also, the temperature was determined when there was no cloud. Based on empirical method of Idso et al. (1981), midday canopy temperature is a suitable indicator to determine the crop water stress. In each plot, the data were prepared from four directions (North, South, East, and West).

**Figure 2:**
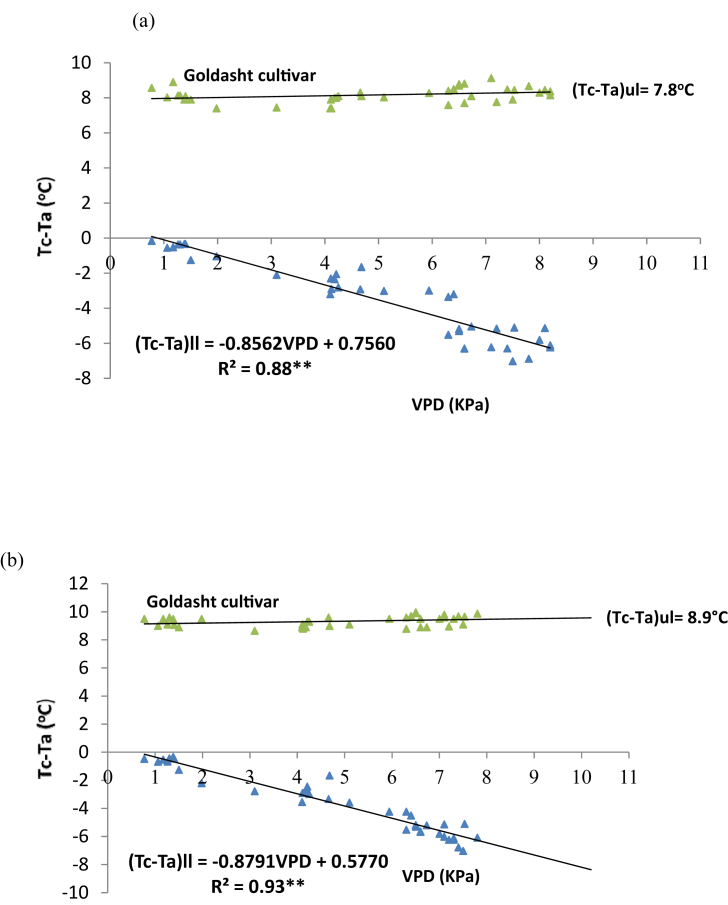
The upper (fully stressed) and lower (non-stressed) baselines for CWSI calculation of Goldasht safflower cultivar during 2017 (a) and 2018 (b) growing seasons. VPD = vapor pressure deficit.

Also, the relative humidity and air temperature were recorded simultaneously at height of 2 m above the ground level, by psychrometer, (Lambrecht, Model 1030, Germany) and a thermo hygrograph (Lambrecht, Model 252, Germany) as a basis for vapor pressure deficit (VPD) determination (Monteith and Unsworth 1990). VPD was determined by standard psychrometer equation (Allen et al., 1998). Then, CWSI amounts were determined by Idso et al. (1981) method, empirically. The relationship between VPD and canopy-air temperature differences (Tc-Ta) was plotted under upper (fully stressed) and lower baselines (non-stressed) equations (Figure 2). In this graph, the non-stressed baseline was evaluated from the data collected three days after irrigation in well-watered treatment.

The non-stressed baseline (lower baseline) can be demonstrated as Eq. (2):

(2) Tc–Ta = aVPD + b

where *a* (slope) and *b* (intercept) are the linear regression coefficients of Tc–Ta on VPD. The upper baseline was determined by the canopy temperatures measured from plots which were kept non-irrigated (fully stressed) between 13:00 to 15:00 h with 30-min intervals.

By the upper and lower limit estimates, a CWSI can be defined using the following Eq. (3) (Idso et al., 1981):

(3) $$\left(\text{CWSI}\right)=\frac{\left(\text{Tc}-\text{Ta}\right)\text{m}-\left(\text{Tc}-\text{Ta}\right)\text{ll}}{\left(\text{Tc}-\text{Ta}\right)\text{ul}-\left(\text{Tc}-\text{Ta}\right)\text{ll}}$$

where (Tc–Ta)m, (Tc–Ta)ll and (Tc–Ta)ul are the determined canopy and air temperature differences at the moment and the lower and upper limit amounts (°C), respectively.

### 2.4 Relative water content (RWC)

The leaf RWC was calculated by Machado and Paulsen's method (2001). In each irrigation regime, at stage 69 [end of flowering; based on BBCH scale illustrated by Flemmer et al. (2015)] eight leaf discs (8 mm in diameter) of expanded leaves around the capitule of main shoot of safflower were weighed for determination of fresh weight (FW). The leaf discs were kept in distilled water for 6 h and after that dried with filter paper and then weighed for determination of total weight (TW). Then, the leaf discs oven dried at 70 °C in 24 h for Dry weight (DW) determination. At last, the RWC was determind by Eq. (4):

(4) RWC = [(FW-DW)/ (TW-DW)] ×100

### 2.5 Plant color quality assessment

The plant color quality in each treatment, was determined according to the Munsell Color Scale (Wilde and Voigt, 1977), from March (before starting the irrigation regimes) up to July (seed maturity stage) during both of the years. In each month, after comparing plant color grades, the page numbers and color were detected based on Munsell Color Scale which is presented in Table 3. In this color scale, dark green color is equal to 9 and yellow color to 1, which showing that the leaf is dead.

**Table 3:** Page number of Munsell Color Chart, color number and visual quality values (Wilde and Voigt, 1977).

Page number of the chart	Color numbers (value/chroma)	Visual quality value	Color changing
5GY	3/4	9	Dark green
5GY	4/4	8	
5GY	4/6, 8	7	
5GY	5/4, 6, 8, 10	6	Green
5GY	6/4, 6, 8, 10	5	
5GY	7/4, 6, 8, 10	4	
2.5GY	7/4, 6, 8	3	
2.5GY	8/4, 6, 8	2	Light green
2.5Y and 5Y	All colors	1	Yellow

### 2.6 Water use efficiency

The water use efficiency (WUE) in each treatment was evaluated as the ratio of seed yield (g. m^−2^) to total water consumed (mm) (Kumar et al., 2019; Howell 2003).

### 2.7 Data analysis

Data was analyzed by SAS software 2012 (version 9.4) and data means in each trait were compared by the least significant differences (LSD) test at 0.05 probability level.

## 3 Results and discussion

### 3.1 Total water consumed

In Goldasht safflower cultivar, total water consumed (irrigation amount + rainfall) in the first year of experiment, were 570.3, 425.5, 283.5, and 140.3 mm for well-watered (100% FC), mild (75% FC), severe (50% FC), and most severe water stress (25% FC), respectively which was less than water consumed by Local Isfahan cultivar (Figure 1). On the other hand, in 2018 growing season, both of the safflower cultivars consumed more water compared to 2017, because of more evaporation and higher mean temperature, especially in reproductive stages of crop (April to Jun) (Table 2). Unfortunately, there was no considerable rainfall in April to June when the water requirement of safflower increased to complete the seed filling period (Table 2). This weather status usually is common in south of Iran and due to occurrence of rain fall in the cool season, the farmers prefer to culture the safflower in December and irrigated the crop in the warm season (Sajedi et al., 2012; Tabib Loghmani et al., 2019).

### 3.2 Upper and lower baselines evaluation

**Figure 3:**
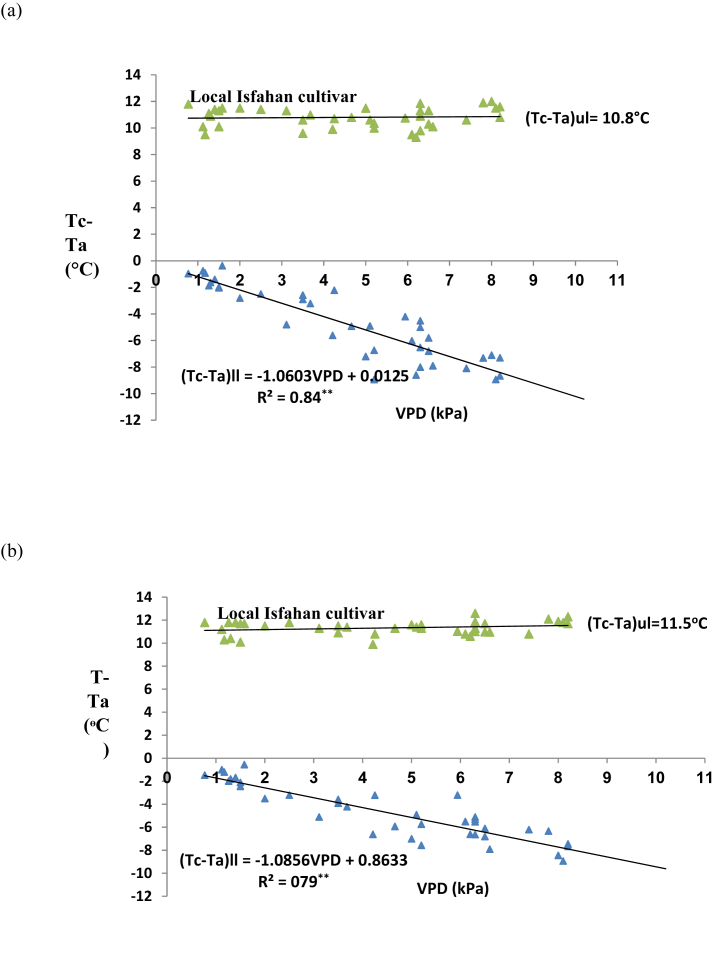
The upper (fully stressed) and lower (non-stressed) baselines for CWSI calculation of Local Isfahan safflower cultivar in 2017 (a) and 2018 (b) growing seasons. VPD = vapor pressure deficit.

In Goldasht safflower cultivar, the upper limit (Tc-Ta)ul, was 7.8 °C in 2017 (Fig. 2a) and 8.9 °C in 2018 (Figure 2b) when air temperature was 37 and 39 °C at solar noon. In two cultivars, by VPD increment due to additional restriction in water availability, the distance between upper and lower base lines increased. Also, the Tc-Ta of lower base line is very important in CWSI determination, practically. The slop (*a*) and intercept (*b*) of lower baseline equation in Goldasht was -0.85 and 0.75 in 2017 (Figure 2a) and -0.87 and 0.57 in 2018, respectively (Figure 2b). In contrast, in Local Isfahan, the upper base line was 10.8 °C (Figure 3a) in 2017 and 11.5 °C in 2018 (Figure 3b) when air temperature was 40 and 42 °C at solar noon. In two years of the experiment, the amount of *a* was in range of -0.85 to -0.87 in Goldasht and -1.06 to -1.08 in Local Isfahan cultivar. It seemed that Local Isfahan safflower cultivars with higher *a* amount had higher sensitivity to VPD increment. Likewise, in Local Isfahan difference between upper base line (fully stressed) and lower base line (non-stressed) was greater than Goldasht cultivar during the 2017 and 2018 growing seasons (Figures 2 and 3). It might be attributed to more sensitivity of Local Isfahan cultivar to water stress which enhances the T*c*-T*a* difference of canopy more than Goldasht cultivar.

Similar to our results Orta et al. (2002) suggested that Tc-Ta determined above a plant canopy was associated to the atmospheric VPD, negatively. Little information has been published concerning the evaluation of lower and upper baselines for CWSI calculation in safflower up to now, while many studies are available in other plants. Sneha et al. (2012) reported that, in mahogany (*Swietenia macrophylla* King). the *a* and *b* amount of lower baseline equation was -0.25 and -2.9, respectively In similar research Emekli et al. (2007) calculated that the upper limit for bermudagrass (*Cynodon dactylon*) was 8.5 °C when air temperature was 40 °C. The slope of lower baseline calculated in India for winter wheat by Gontia and Tiwari (2008) which was close to our data (−0.85 to -1.08). Likewise, in south of Iran, Irandoust and Bijanzadeh (2018) declared a slope amount of -1.0 and an intercept of 0.72 in wheat. Generally, differences in the lower and upper baseline equations could be related to crop type, plant density, crop color quality, volume of water consumed and rainfall amount during the reproductive stages (Bastug and Buyuktas, 2003; Alghory and Yazar, 2019; Bijanzadeh et al., 2019).

### 3.3 CWSI assessment

According to the climatic data of the both years (Table 2), irrigation treatments were started by cutting off effective irrigation from April up to late season in the June and for this reason the mean monthly and seasonal CWSI amounts of safflower cultivars under different irrigation regimes were calculated during the April to July of 2018 (2017-18 growing season) and 2019 (2018-19 growing season) (Table 4). In both of the growing seasons and each irrigation regime, Goldasht cultivar had lower amount of monthly CWSI compared to Local Isfahan. By increasing the air temperature and evaporation from April to June, CWSI increased in Local Isfahan more than Goldasht, significantly (p ≤ 0.05). In the first year, CWSI amount showed an increasing trend from April (0.071) in well-watered of Goldasht to July (0.831) in most severe water stress treatment of Local Isfahan because of increment in Tc-Ta differential and higher VPD amounts (Figures 2 and 3) during the reproductive stage of safflower (Table 4). Interestingly, the same trend was observed in 2018 growing season but CWSI amount in 2018 was more than 2017 in both of the safflower cultivars, because of higher evaporation and temperature during April to July in comparison to 2017 growing seasons (Table 2). In second year, CWSI in April from 0.097 in well-watered of Goldasht reached to 0.901 in July under most severe water stress of Local Isfahan, significantly (p ≤ 0.05). When the available water content is limited under stress, VPD was increased and crop transpiration restricted which caused Tc-Ta differential enhancement (Emekli et al., 2007; Bijanzadeh et al., 2019; Kumar et al., 2019). In the current study, in two years of experiment, as air warmed from April to July (Table 2), Tc-Ta differential was enhanced (Figures 2 and 3) and the highest monthly mean amount of CWSI were observed in July (Table 4).

**Table 4:** Mean monthly and seasonal CWSI amounts of safflower cultivars under different irrigation regimes in 2017–2018 and 2018–2019 growing seasons.

Irrigation regimes	Cultivar type	Mean				CWSI				Mean Seasonal CWSI	
		April		May		June		July			
		2017–18	2018–19	2017–18	2018–19	2017–18	2018–19	2017–18	2018–19	2017–18	2018–19
Well-watered	Goldasht	0.071	0.097	0.124	0.217	0.244	0.266	0.267	0.291	**0.18**	**0.22**
	Local Isfahan	0.084	0.112	0.131	0.231	0.256	0.278	0.277	0.311	**0.19**	**0.23**
Mild water stress	Goldasht	0.125	0.139	0.291	0.31	0.323	0.386	0.364	0.381	**0.28**	**0.30**
	Local Isfahan	0.133	0.185	0.315	0.343	0.366	0.401	0.389	0.397	**0.30**	**0.33**
Severe water stress	Goldasht	0.486	0.511	0.496	0.581	0.62	0.659	0.674	0.691	**0.57**	**0.61**
	Local Isfahan	0.514	0.556	0.544	0.613	0.672	0.679	0.721	0.795	**0.61**	**0.66**
Most severe water stress	Goldasht	0.514	0.556	0.611	0.687	0.643	0.714	0.701	0.784	**0.62**	**0.69**
	Local Isfahan	0.623	0.657	0.698	0.745	0.745	0.789	0.831	0.901	**0.72**	**0.77**
LSD (0.05)		0.09	0.11	0.09	0.1	0.13	0.09	0.15	0.11	**0.12**	**0.10**

Results showed that mean seasonal CWSI amounts in second year was more than the first year due to higher mean temperature and evaporation from April to July of the 2018 growing season (Table 2), and in Local Isfahan was higher than Goldasht which demonstrated the susceptibility of Local Isfahan to water stress. Under the most severe water stress treatments, Local Isfahan by 0.72 and 0.77 amounts had the highest mean seasonal CWSI in 2017 and 2018, respectively (Table 4). In 2017 and 2018 growing seasons, differences between mean CWSI amount of well-watered with severe (50%FC) and most severe water stress (25% FC) treatments was significant (*p* ≤ 0.05). In Iran, safflower is usually grown at the same time as wheat and canola. In similar study, Alghory and Yazar (2019) reported that when wheat irrigated only at flowering, the CWSI amount increased after flowering up to seed maturity stage and this increment might be attributed to canopy temperatures increasing in late season and irrigation cut off at flowering which led leaf dried and promote plant senescing; as a result, canopy temperature became higher and caused CWSI increment. They concluded that the CWSI amount for winter wheat in the mild water-stressed conditions was ranged 0.31 to 0.41 in dry years and 0.25 to 0.32 in wet years which might be determined as deficit irrigation regime. Asemanrafat and Honar (2017) declared that in red bean (*Phaseolus vulgaris* L. CV. Akhtar) CWSI values in mild water stress [irrigation according to 80% evapotranspiration (ET)] varied from 0.14 to 0.44. Fattahi et al. (2018) showed that a and b in the lower baseline equation in irrigation according to 75% total available water (TAW) was -1.41 and -1.7 for corn, respectively and upper baseline equation was 2.3 °C. They concluded that irrigation scheduling in corn field should be done by 75% TAW. Kumar et al. (2019) showed that in Indian mustard (*Brassica juncea*) in sever water stress (50% SMD), the CWSI kept above 0.3 for most part of the growing season and maximized to 0.9 at maturity stage. Heydari et al. (2019) suggested that in semi-arid regions, with respect to yield production and RWC, irrigation according to 75% FC in canola (*Brassica napus* L.) is a suitable strategy for canola while CWSI amount ranged from 0.198 to 0.294. As mentioned in the previous researches above, when the CWSI amount is closer to zero, the crop demonstrated the better performance. Therefore, with respect to water deficit at reproductive stage of safflower in southern Iran (Table 2), the mild water stress (75% FC) in the present study could be a suitable irrigation regime which mean CWSI amount was in range of 0.28–0.33 compared to severe and the most severe water stress which ranged 0.57 to 0.77 (Table 4).

### 3.4 RWC evaluation

**Figure 4:**
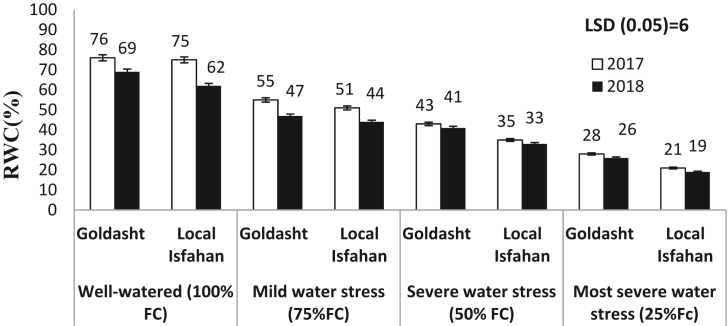
Effect of irrigation regime on RWC of safflower cultivars in 2017 and 2018 growing seasons. Vertical bar represents ±SE.

The leaf relative water content (RWC) is a key index in evaluation of water conditions of leaves especially when crop subjected to water stress. During the both years, RWC from well watered treatment to most sever water stress decreased, significantly (p ≤ 0.05) and in Local Isfahan was less than Goldasht safflower cultivar (Figure 4). Under water stress treatments, the highest RWC (55%) was obtained in Goldasht in 2017 growing seasons when plant exposed to mild water stress and reached to 28% in most severe water stress (49% reduction), while in this year in Local Isfahan from 51% in mild water stress reached to 21% in most severe water stress (58% reduction). Pasban Eslam (2011) declared that in safflower (CV. Local Isfahan) RWC from 76% in non-stressed treatments declined, significantly to 62% in water stress subjected from 80%flowering to seed maturity. Tabib Loghmani et al. (2019) declared that RWC influenced by the main effects of crop genotype, irrigation regime, and year, and interaction of genotype and irrigation regime, significantly (p ≤ 0.01). Similar to our results they declared that water stress caused RWC declined in comparison to well-watered irrigation regime, significantly. In our study, RWC in Goldasht was maintained higher than Local Isfahan especially when subjected to high levels of water stress (50–25% FC). Overall, during the both years, Goldasht safflower cultivar had the lower CWSI (Table 4) which should be attributed to maintain the higher amount of RWC (Figure 4) in their leaves compared to Local Isfahan.

### 3.5 Plant color quality

At late March (initial stage of measurements), before the safflower were subjected to water stress [Stage 55; based on BBCH scale illustrated by Flemmer et al. (2015)], there was the same color grading (8) for all irrigation regimes (Table 5). Actually, in 2017 and 2018 growing season up to March, the soil water volume in all irrigation regimes was adequate for safflower growth due to suitable rainfall (Table 2). Based on Munsell Color Scale described by Wilde and Voigt (1977) an acceptable color quality was in range of score 9 (dark green) to around the score 6 (green) and below 6 the color quality decreased up to score 1 (yellow color) which is plant death stage (Table 3). During the both years of experiment, in unirrigated plot, from May to July the color quality stayed constant and reached to 1, sharply due to no available water in the soil. In April, by improving water stress level, the score of leaf color declined from 7 to 2 and this similar trend was continued up to reach to 1 at seed maturity stage in July. Also, the color grading score in Local Isfahan safflower cultivar was lower than Goldasht and in second year was less than first year. It might be attributed the more tolerance of Goldasht to water stress, while mean air temperature and evaporation in 2017 was less than 2018 growing season (Table 2). During the two growing seasons when plants exposed to different irrigation regimes, in Goldasht the mean seasonal color quality from 6.4 reached to 3.4 while in Local Isfahan from 5.2 declined to 2.6. Bijanzadeh et al. (2019) declared that in bur clover (*Medicago polymorpha* L.), the grade of color in the unirrigated plot was declined, sharply from 8 to 1 and crop died, completely in this plot by the late July. Karcher and Richardson (2003) found that in turfgrass (*Cynodon dactylon* L.) the score of color was in range from 9 to 1 during the growth season, while grade 6 was the minimum acceptable grade for this grass. Also, Bastug and Buyuktas (2003) suggested that in turfgrass, the suitable color quality under the Mediterranean climate might be gained as irrigation water was consumed as much as 75% of evaporation. Bonos and Murphy (1999) reported that water stress due to hot days in summer can influence the color quality of Kentucky bluegrass (*Poa pratensis*), negatively. In the current study, in Goldasht an acceptable color quality between 6.4 to 5.6 was achieved in April and May, under well-watered and mild water stress conditions (75% FC), but the mean color quality gained in most severe water stress (25% FC) were not suitable (3.6–3.0) especially in Local Isfahan during the 2017 and 2018 growing seasons.

**Table 5:** Visual plant color quality values of safflower during the experiment under different irrigation regimes in 2017–2018 and 2018–2019 growing seasons.

Irrigation regimes						Visual color		quality values						
	Cultivar type	Initial (before stress)		April		May		June		July		Mean Seasonal		
		2017–18	2018–19	2017–18	2018–19	2017–18	2018–19	2017–18	2018–19	2017–18	2018–19	2017–18	2018–19	
Well-watered	Goldasht	8	8	7	7	6	6	5	5	6	6	6.4	6.4	
	Local Isfahan	8	8	6	6	5	5	4	4	3	3	5.2	5.2	
Mild water stress	Goldasht	8	8	7	6	6	5	4	4	6	5	6.2	5.6	
	Local Isfahan	8	8	5	5	4	3	3	3	3	2	4.6	4.2	
Severe water stress	Goldasht	8	8	5	5	4	3	3	3	3	2	4.6	4.4	
	Local Isfahan	8	8	4	3	3	3	3	2	2	2	4.0	3.6	
Most severe water stress	Goldasht	8	8	3	3	3	2	2	2	2	2	3.6	3.4	
	Local Isfahan	8	8	3	2	2	2	2	2	1	1	3.2	3.0	
Unirrigated	Goldasht	8	8	4	2	1	1	1	1	1	1	3.0	2.6	
	Local Isfahan	8	8	3	1	1	1	1	1	1	1	2.8	2.4	

### 3.6 Seed yield production

**Figure 5:**
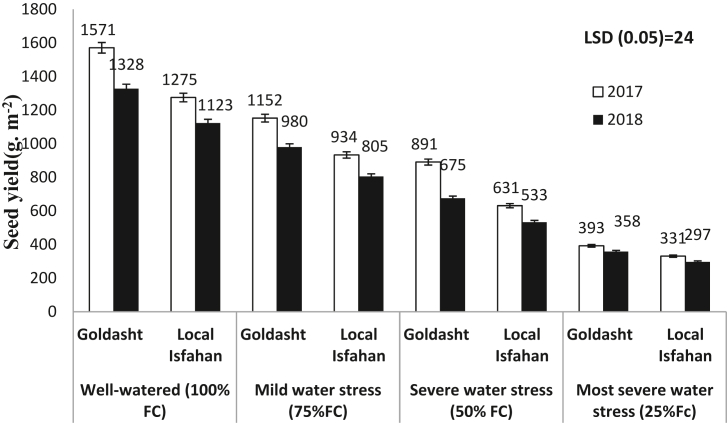
Effect of irrigation regime on seed yield production of safflower cultivars in 2017 and 2018 growing seasons. Vertical bar represents ±SE.

The seed yield production was sensitive to water stress, so that well-watered treatment had the highest seed yield, in 2017 and 2018 growing seasons, significantly (p ≤ 0.05) (Figure 5). In each irrigation regime and safflower cultivar, seed yield in 2017 was more than 2018, significantly. It might be related to higher CWSI amount in 2018 in comparison to 2017 growing season from April to July (Table 4). Also, in 2017, seed yield of Goldasht, in well-watered, mild, severe and the most severe water stress was 1571, 1152, 891, and 393 g m^−2^ which was 23, 20, 41 and 18% more than Local Isfahan, respectively. Similar trend was observed in these treatments in 2018, and seed yield in Local Isfahan from 1123 g m^−2^ declined to 805, 533 and 297 g m^−2^, significantly (*p* ≤ 0.05) (Figure 5). Zareie et al. (2013) reported that for adaptation of safflower to water stress needs improved tolerance to water shortage in flowering stage. Our results showed that in all water stress levels, seed yield in Goldasht was more than Local Isfahan and in might be related to better adaptation of this cultivar to water stress conditions from flowering to seed maturity stages compared to Local Isfahan. It is appeared water stress in reproductive period with decreasing the flowering duration and florets infertility caused seed yield loss. Omidi et al. (2012) reported that in safflower (cv. Padideh) seed yield was the only trait significantly influenced when irrigation was cut off at seed filling, compared to well-watered treatment. Also, irrigation termination at early flowering produced only 136 g m^−2^ seed yield in comparison to 219 g m^−2^ in well-watered (61% reduction). Pasban Eslam (2011) reported that in safflower (CV. Local Isfahan) seed yield from 216.4 in well-watered reached to 178.1 kg ha^−1^ (21% reduction) in cut off irrigation from 80% flowering to seed maturity. Sajedi et al. (2012) declared that, in irrigation based on 100 %, 75% and 50% FC, the safflower seed yields of Local Isfahan cultivar were 1583, 1341 and 1284 g m^−2^, respectively. Tabib Loghmani et al. (2019) declared that safflower seed yield declined sharply, in cut off irrigation from bud formation to maturity, significantly (*p* ≤ 0.05). In our study, in the both years, seed yield production was in range of 1571 to 297 g m^−2^ (Figure 5). Also, our results declared that water stress increased yield loss compared to well-watered especially in Local Isfahan and this depletion could be attributed to biological aging and hastening of senescence due to cut off irrigation which reduced leaf color quality (Table 5) and seed yield (Figure 5). Overall, applying a mild water stress according to 75%FC saved approximately 25% water which safflower yield reduction was in range of 26.1%–28.3% in comparison to well-watered condition. However, this treatment can be recommended under water deficient condition in semi-arid areas, where the seed yield penalty for deficit irrigations is minimized.

### 3.7 WUE

**Figure 6:**
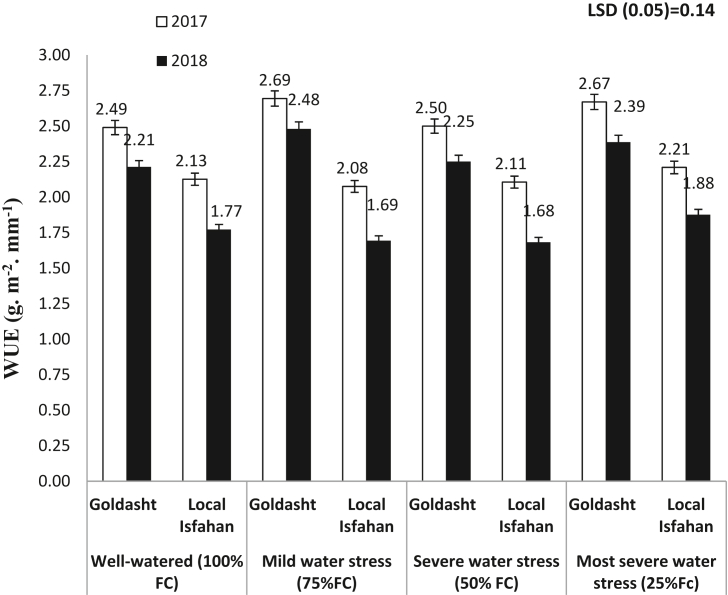
Effect of irrigation regime on WUE of safflower cultivars in 2017 and 2018 growing seasons. Vertical bar represents ±SE.

Water use efficiency in each irrigation regime and safflower cultivar in 2017 and 2018 are presented in Figure 6. Results showed that in all of the irrigation regimes WUE in 2017 was higher than 2018 mainly in Local Isfahan compared to Goldasht. Interestingly, in 2017, the highest WUE was 2.69 and 2.67 g m^−2^ mm^−1^ in mild and most severe water stress treatments of Goldasht cultivar, respectively. Similar results were observed in 2018 when a slightly lower maximum WUE of 2.48 and 2.39 g m^−2^ mm^−1^ was recorded in these treatments. During the both years and cultivars, WUE in most severe water stress (25% FC) was more than severe water stress (50%FC) which may be due to higher water consumed in severe water stress compared to most sever water stress treatment (Figure 1). Alghory and Yazar (2019) in a study on wheat declared that the highest value of WUE (1.35 kg m^− 3^) was obtained in irrigation according to 75% conventional irrigation in comparison to 25 and 50% irrigation levels. Our results are in agreement to a number of studies suggesting that water stress and deficit irrigation, especially during the reproductive growth stages could be increased WUE, significantly (Rao et al., 2012; Irandoust and Bijanzadeh, 2018). Kumar et al. (2019) declared that in Indian mustard although well-watered treatment created 900 kg ha^−1^ seed yield, but consumed 260 mm irrigation water. In contrast, irrigation regime corresponding to 30% soil moisture depletion (SMD) gained 850 kg ha^−1^ seed yield, while only consumed 200 mm irrigation water and had the highest WUE. In the present study, WUE of mild water stress (75%FC) was more than well-watered treatment (100% FC) which indicated that the optimal irrigation treatment to reach acceptable seed yield and water consumption. In other words, with respect to WUE, mild water stress treatment by 25% water saving produced more seed yield compared to well-watered conditions.

### 3.8 Relationship between CWSI and other traits

**Figure 7:**
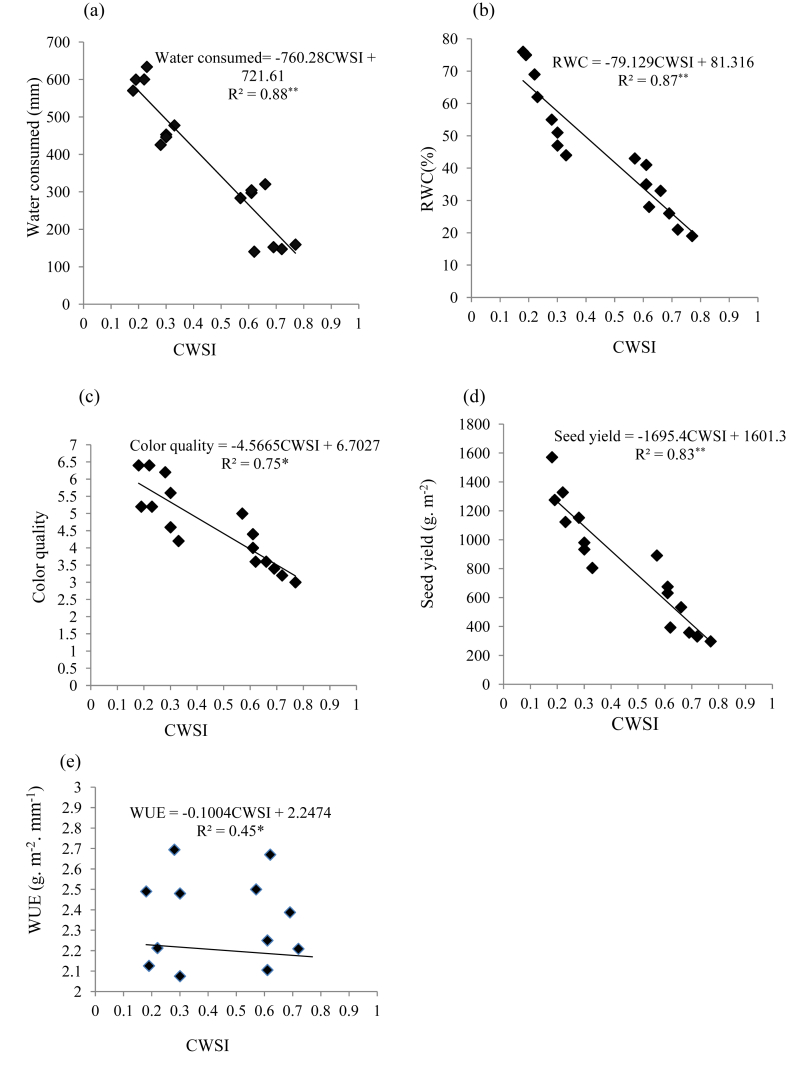
Relationship between CWSI with water consumed (a), RWC (b), color quality (c), seed yield (d), and WUE (e) of safflower in 2017 and 2018 growing seasons.

The interrelationship between CWSI and total water consumed during the growing season are presented in Figure 7a by a linear regression. Results declared that by declining water consumption in range of 570–633 mm in well-watered to 159-140 mm in most severe water stress, CWSI was increased from 0.18 to 0.77 sharply so that, CWSI was correlated with water consumed, negatively (R^2^ = 0.88∗∗). A negative and linear relationship was obtained between CWSI and RWC, by this equation:

RWC = -79.129CWSI+81.316 Also, by decreasing the RWC from 43% to 19%, CWSI increased, sharply (Figure 7b). The color quality grades were affected by CWSI changes during the growth season, negatively (R^2^ = 0.75∗), so that the higher score was obtained in lower CWSI (Figure 7c). By increasing CWSI from 0.18 to 0.77 grain yield declined from 1571 to 297 g m^−2^, negatively (R^2^ = 0.83∗∗).

On the hand, by this linear equation:

Seed yield = -1695.4CWSI+1601.3

where seed yield of safflower could be predicted of by CWSI assessment (Figure 7d). Also, with CWSI increment the WUE decreased, slowly (R^2^ = 0.45∗) (Figure 7e). Asemanrafat and Honar (2017) declared that in red bean CWSI had negative and significant (*p* ≤ 0.01) correlation to grain yield, represented that grain yield decreased with CWSI increment. Also, the negative interrelationship between CWSI and grain yield was declared by Wang et al. (2005), Orta et al. (2002), Zia et al. (2012). Irandoust and Bijanzadeh (2018) observed that canopy color quality correlated to CWSI, negatively. Overall, an accurate correlation by a linear equation between CWSI and seed yield can be applied for yield prediction (Bijanzadeh et al., 2019; Alghory and Yazar, 2019). In the present study, interrelationship between CWSI and other traits declared that the slope of the relationships between CSWI and water consumed, RWC, color quality, and seed yield, respectively, was negative, showing that by increasing the CWSI these traits reduced significantly. On the other hand, a negative relationship was observed between CWSI and WUE, while the slope of the equation was less than the other traits (Figure 7).

## 4 Conclusions

In the present study, CWSI was determined in four different irrigation regimes based on FC in two safflower cultivars in Darab during 2017 and 2018, growing seasons. In Goldasht cultivar, the upper limit (Tc-Ta)ul, was 7.8 °C in 2017 and 8.9 °C in 2018 when air temperature was 37 and 39 °C at solar noon. Local Isfahan cultivar consumed more water and had less RWC, color quality and WUE compared to Goldasht which caused higher CWSI when plants exposed to water stress. It might be attributed the more tolerance of Goldasht to water deficit and lower air temperature and evaporation during 2017 growing season. During the both of the experiment, a negative interrelationship was gained between CWSI and these traits and its equation could be predicted seed yield of safflower. We concluded that irrigation according to 75% FC, when CWSI was in range of 0.28–0.33 could be an acceptable irrigation regime for safflower under water deficit conditions.

## Declarations

### Author contribution statement

Ehsan Bijanzadeh: Analyzed and interpreted the data; Wrote the paper.

Seyed Mojtaba Moosavi: Performed the experiments; Contributed reagents, materials, analysis tools or data.

Fatemeh Bahadori: Conceived and designed the experiments; Performed the experiments.

### Funding statement

This work was supported by 10.13039/501100005071Shiraz University, Shiraz, Iran.

### Data availability statement

Data associated with this study has been deposited at 10.5281/zenodo.5916168.

### Declaration of interests statement

The authors declare no conflict of interest.

### Additional information

No additional information is available for this paper.
